# Sox2 modulates the function of two distinct cell lineages in mouse skin^☆^

**DOI:** 10.1016/j.ydbio.2013.08.004

**Published:** 2013-10-01

**Authors:** Marta H. Lesko, Ryan R. Driskell, Kai Kretzschmar, Stephen J. Goldie, Fiona M. Watt

**Affiliations:** a Wellcome Trust – Medical Research Council Centre for Stem Cell Institute, University of Cambridge, Tennis Court Road, Cambridge CB2 1QN, UK; b Cancer Research UK Cambridge Research Institute, Robinson Way, Cambridge CB2 0RE, UK; c Centre for Stem Cells and Regenerative Medicine, King's College London, 28th Floor, Guy's Tower, London SE1 9RT, UK

**Keywords:** Merkel cell, Dermal papilla, Stem cell

## Abstract

In postnatal skin the transcription factor Sox2 is expressed in the dermal papilla (DP) of guard/awl/auchene hair follicles and by mechanosensory Merkel cells in the touch domes of guard hairs. To investigate the consequences of Sox2 ablation in skin we deleted Sox2 in DP cells via Blimp1Cre and in Merkel cells via K14Cre. Loss of Sox2 from the DP did not inhibit hair follicle morphogenesis or establishment of the dermis and hypodermis. However, Sox2 expression in the DP was necessary for postnatal maintenance of awl/auchene hair follicles. Deletion of Sox2 via K14Cre resulted in a decreased number of Merkel cells but had no effect on other epithelial compartments or on the dermis. The reduced number of Merkel cells did not affect the number or patterning of guard hairs, nerve density or the interaction of nerve cells with the touch domes. We conclude that Sox2 is a marker of two distinct lineages in the skin and regulates the number of differentiated cells in the case of the Merkel cell lineage and hair follicle type in the case of the DP.

## Highlights

• Sox2 is a marker of two distinct lineages in the skin.
• Sox2 is required for postnatal maintenance of awl/auchene hair follicles.
• Loss of Sox2 results in a reduction in Merkel cells.

## Introduction

The transcription factor Sox2 is involved in maintenance of the early, pluripotent stem cells of the eipiblast (Avilion et al., 2003) and in re-establishing pluripotency in postnatal cell types (Takahashi and Yamanaka, 2006). Sox2 is essential for central nervous system (CNS) development and maintenance of neural stem cells (Pevny and Nicolis, 2010). Sox2 is also expressed in adult stem cells and progenitors and plays a crucial role in tissue regeneration in various organs (Arnold et al., 2011).

Sox2 is expressed in the dermal papilla cells of guard/awl/auchene hair follicles (Driskell et al., 2009) and in the dermal sheath cells of some hair follicles (Laga et al., 2010). Dermal papillae are specialised clusters of fibroblasts at the base of each hair follicle that regulate follicle development and cycling via reciprocal signalling with the overlying epidermal cells (Millar, 2002; Driskell et al., 2011). Depletion of Sox2-positive DP cells prevents formation of awl/auchene hair follicles in skin reconstitution assays (Driskell et al., 2009). When Sox2-positive dermal cells are cultured and subsequently grafted into mice they retain their identity, suggesting that they represent a distinct dermal lineage (Driskell et al., 2012b). In those assays Sox2-positive cells not only contribute to the DP but can also be more widely distributed in the dermis (Driskell et al., 2012b), consistent with previous reports that Sox2-positive dermal cells are multipotent Skin Derived Precursors (SKPs) (Toma et al., 2001; Fernandes et al., 2004; Biernaskie et al., 2009).

Within the epidermis Sox2 is expressed in a small population of mechanosensory cells known as Merkel cells (Haeberle et al., 2004; Driskell et al., 2009). These neuroendocrine cells are clustered in the epidermal basal layer adjacent to guard hairs, and constitute touch domes (Lumpkin and Caterina, 2007; Lumpkin et al., 2010). Merkel cells are excitable, express voltage-gated ion channels and are capable of calcium-induced calcium release (Piskorowski et al., 2008; Haeberle, 2004). They also express simple keratins (K8, 18 and 20), neuropeptides and presynaptic machinery proteins (such as Rab3c), as well as transcription factors involved in neuronal cell fate determination (Haeberle et al., 2008). Merkel cells are postmitotic, terminally differentiated cells that are derived from keratin 14-positive cells in the epidermal basal layer that downregulate keratin 14 on differentiation (Van Keymeulen et al., 2009; Woo et al., 2010; Morrison et al., 2009).

In view of the key contributions of DP cells and Merkel cells to skin function and the observation that Sox2 is a marker of SKPs, we have investigated the consequences of deleting Sox2 in the DP and Merkel cell compartments.

## Material and methods

### Transgenic mice

All experiments were approved by King's College London, Cambridge University and Cancer Research UK local ethics committees and performed under the terms of a UK government Home Office licence. Sox2fl/fl mice, in which flox sequences flank the Sox2 locus (Favaro et al., 2009), were kindly provided by Silvia Nicolis. CAGCATeGFP, Blimp1Cre and Blimp1GFP mice have been described previously (Kawamoto et al., 2000; Ohinata et al., 2005). NOD.Cg-Prkdcscid Il2rgtm1Wjl/SzJ (NSG) immunodeficient mice were acquired from the Jackson Laboratory. K14Cre mice were a kind gift of Michaela Frye (Driskell et al., 2012a) and were originally obtained from the Jackson Laboratory.

### Flow cytometry

Flow cytometry was performed on dermal preparations as described previously (Jensen et al., 2010) using a Cyan Flow Analyser. CD133-APC (eBiosciences) and eCadherin-647 antibodies (eBiosciences) were used at the manufacturer's recommended concentrations. Analysis of flow cytometry data was performed using FlowJo software.

Gating criteria were as follows. Debris was gated out using forward and side scatter plots. Doublets and dead cells were also gated out and analysis was performed on live cells using GFP and APC channels. Gating for positively labelled cells was performed against negative control samples to less than 0.5% background.

### Histology, whole mounts and immunostaining

Preparation and immunostaining of conventional cryosections (5–30 μm thick) and whole mounts of tail epidermis, back skin and whisker pad were performed as described previously (Driskell et al., 2009). Back skin horizontal whole mounts (100 μm thick) were prepared and immunostained as described by Driskell et al. (2012b).

The following primary antibodies were used at the dilutions indicated: Sox2 1:100 (R&D Systems), CD133 1:50 (eBioscience), Itga8 1:200 (R&D Systems), Dcc 1:100 (R&D Systems), K14 1:1000 (Covance), Blimp1 1:50 (eBioscience), Corin 1:100 (R&D Systems), K8 1:100 (developed by P. Brulet and R. Kemler, and obtained from the Developmental Studies Hybridoma Bank developed under the auspices of the NICHD and maintained by The University of Iowa, Department of Biology, Iowa City, IA 52242), NF-H 1:100 (Millipore), PGP9.5 1:100 (Dako), V-GLUT2 1:500 (Invitrogen), Rab3c 1:3000 (Abcam), synapsin II 1:200 (Abcam), Cav2.1 1:100 (Millipore), piccolo 1:2000 (SYSY) and GFP 1:500 (Invitrogen).

### Nerve density quantification

Images of back skin whole mounts immunostained for PGP9.5 were acquired from the epidermal side using a confocal microscope. The Metamorph software tube formation application was used to quantitate the percentage of total skin area covered by nerves.

### Transmission electron microscopy

Back skin was collected from E18.5 mice. Sample processing and image capture were carried out by Dr Jeremy Skepper, Anatomy Department, Multi-Imaging Centre, University of Cambridge. Tissues were fixed in 4% glutaraldehyde in 0.1 M HEPES buffer at pH 7.4 for 12 h at 4 °C and rinsed 5 times in 0.1 M HEPES buffer. Tissues were then treated with 1% osmium ferricyanide at room temperature for 2 h, rinsed 5 times in distilled water and treated with 2% uranyl acetate in 0.05 M maleate buffer at pH 5.5 for 2 h at room temperature. Tissues were rinsed in distilled water and dehydrated in an ascending series of ethanol solutions from 70% to 100%. This was followed by treatment with two changes of dry acetonitrile and infiltration with Quetol epoxy resin. Images were taken with a FEI Tecnai G2 operated at 120 Kv using an AMT XR60B digital camera running Deben software.

### Embryonic skin grafting

E18.5 embryos were harvested from Blimp1Cre+/Sox2fl/wt×Sox2fl/fl intercrosses. Skin was dissected from the embryos and surgically sutured onto the backs of adult NSG mice. Sutures were removed after 1 week and grafts were harvested after 10 weeks.

### Statistical analysis

The number of Merkel cells per touch dome was quantitated in 7 mice per genotype. For each biological replicate 3 areas were analysed, corresponding to a total of 30 touch domes per mouse. Nerve density was analysed in 8 biological replicates, with 3 areas per mouse. Innervation of Merkel cells (10 touch domes in each biological sample), touch domes per unit area and guard hairs per unit area were analysed in 4 biological replicates per genotype. In all cases data from the biological replicates were pooled. The D’Agostino and Pearson omnibus test was used to examine normality of data distribution. Since none of the data followed a Gaussian distribution, a non-parametric Mann-Whitney test was applied to examine the significance of differences between datasets.

## Results

### Blimp1 is expressed in the dermal condensate during skin morphogenesis

In order to delete Sox2 in the DP we required a promoter that would drive Cre expression in the dermis at the appropriate stages of development. One candidate was the promoter of the transcription factor B lymphocyte induced maturation protein (Blimp1). Blimp1 has previously been shown to be required for maintenance but not initial specification of DP progenitors (Horsley et al., 2006; Robertson et al., 2007).

**Fig. 1:**
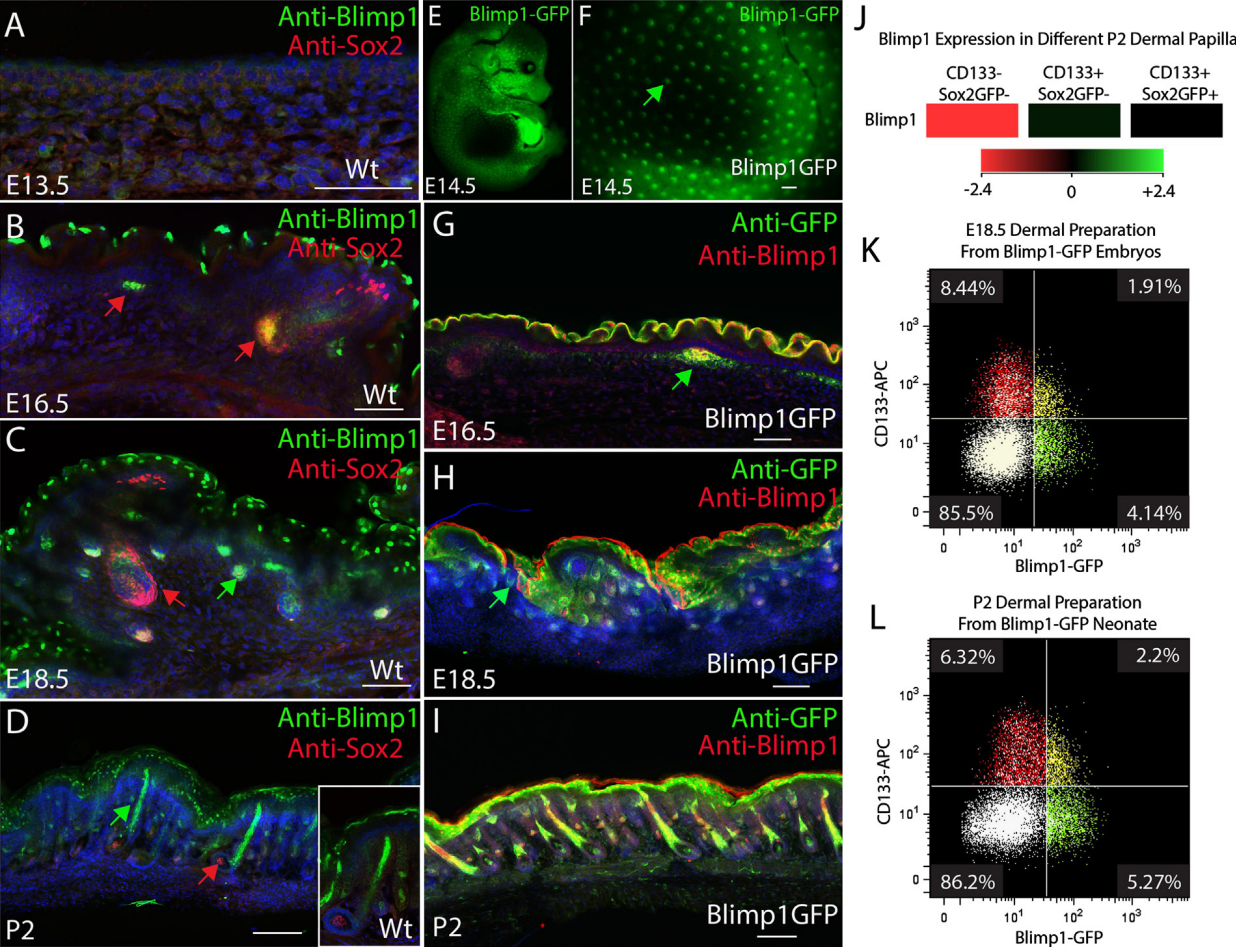
Blimp1 is expressed by dermal papilla cells during skin morphogenesis. (A–D) Cryosections of E13.5-P2 skin labelled with antibodies to Sox2 and Blimp1. Note Blimp1 expression in DP of guard/awl/auchene hair follicles (red arrows) and zigzag hair follicles (green arrows) and in the outermost epidermal cell layers (B–D). (E–I) GFP expression under the control of Blimp1 regulatory elements colocalizes with endogenous Blimp1 during morphogenesis of guard/awl/auchene (E–G) and zigzag (H–I) hairs. Arrows show dermal condensates (F, G) and dermal papilla (H). (J) Blimp1 mRNA was detected in guard/awl/auchene (CD133+Sox2GFP+) and zigzag (CD133+Sox2GFP−) dermal papilla cells, but not in dermal fibroblasts (CD133−Sox2GFP−). (K, L) Flow cytometry of single cell suspensions of E18.5 (H) and P2 (I) dermal cells, showing co-expression of the dermal papilla marker CD133 with Blimp1GFP. Cells in each quadrant are labelled with a different colour for ease of visualisation. Data are representative of at least *N*=3 biological replicates. Scale bars: (A, B) 1 mm, (C–G) 100 μm.

To examine Blimp1 expression in developing skin we performed immunostaining with Blimp1 antibodies and also examined expression of GFP under the control of the Blimp1 transcriptional regulatory regions (Ohinata et al., 2005). Blimp1 was not detected in E13.5 skin (Fig. 1A and data not shown). However, it was expressed at the onset of hair follicle morphogenesis at E14.5 in the precursors of the dermal papillae (the dermal condensate) of all types of hair follicle, including Sox2-positive DP (Fig. 1B, C, E–H), consistent with previous reports (Horsley et al., 2006; Robertson et al., 2007). There was no detectable difference in Blimp1 levels between different hair follicle types. At P2 Blimp1 continued to be expressed in the dermal papillae of all follicles, albeit at lower levels than in the DP precursors (Fig. 1D and I). We confirmed that GFP expression overlapped with endogenous Blimp1 (Fig. 1G–I). Blimp1 expression was not confined to the dermis and from E16.5 onwards there was a progressive increase in Blimp1 expression in the suprabasal layers of the interfollicular epidermis, consistent with previous reports (Magnusdottir et al., 2007). In contrast, Sox2 is not expressed by differentiating epidermal cells of the interfollicular epidermis (Driskell et al., 2009).

Analysis of Blimp1 mRNA expression levels in previously published microarray datasets (Driskell et al., 2009) revealed that guard/awl/auchene (CD133+Sox2GFP+) and zigzag hair dermal papilla (CD133+Sox2GFP−) cells express higher levels of Blimp1 than non-DP dermal cells (CD133−Sox2GFP−) (Fig. 1J). We isolated dermal cells from E18.5 and P2 dermis of Blimp1GFP mice and compared expression of GFP and the DP marker CD133 (Ito et al., 2007; Driskell et al., 2009) (Fig. 1K and L). We confirmed that there were no GFP+ eCadherin+ cells in the preparation, excluding the possibility of epidermal contamination (data not shown). Three distinct cell populations were observed: CD133+GFP+, CD133+GFP− and CD133−GFP+. The existence of the CD133−GFP+ population is consistent with Blimp1 being expressed both in DP and non-DP fibroblasts (Robertson et al., 2007). The existence of CD133+GFP− cells suggests that there are Blimp1− cells in the DP and most likely reflects the variation in developmental stage of individual DPs at the time-points examined, since Blimp1 expression is highest early in DP development, while CD133 expression is highest at later stages.

### Blimp1 defines dermal condensates of all hair follicle types

**Fig. 2:**
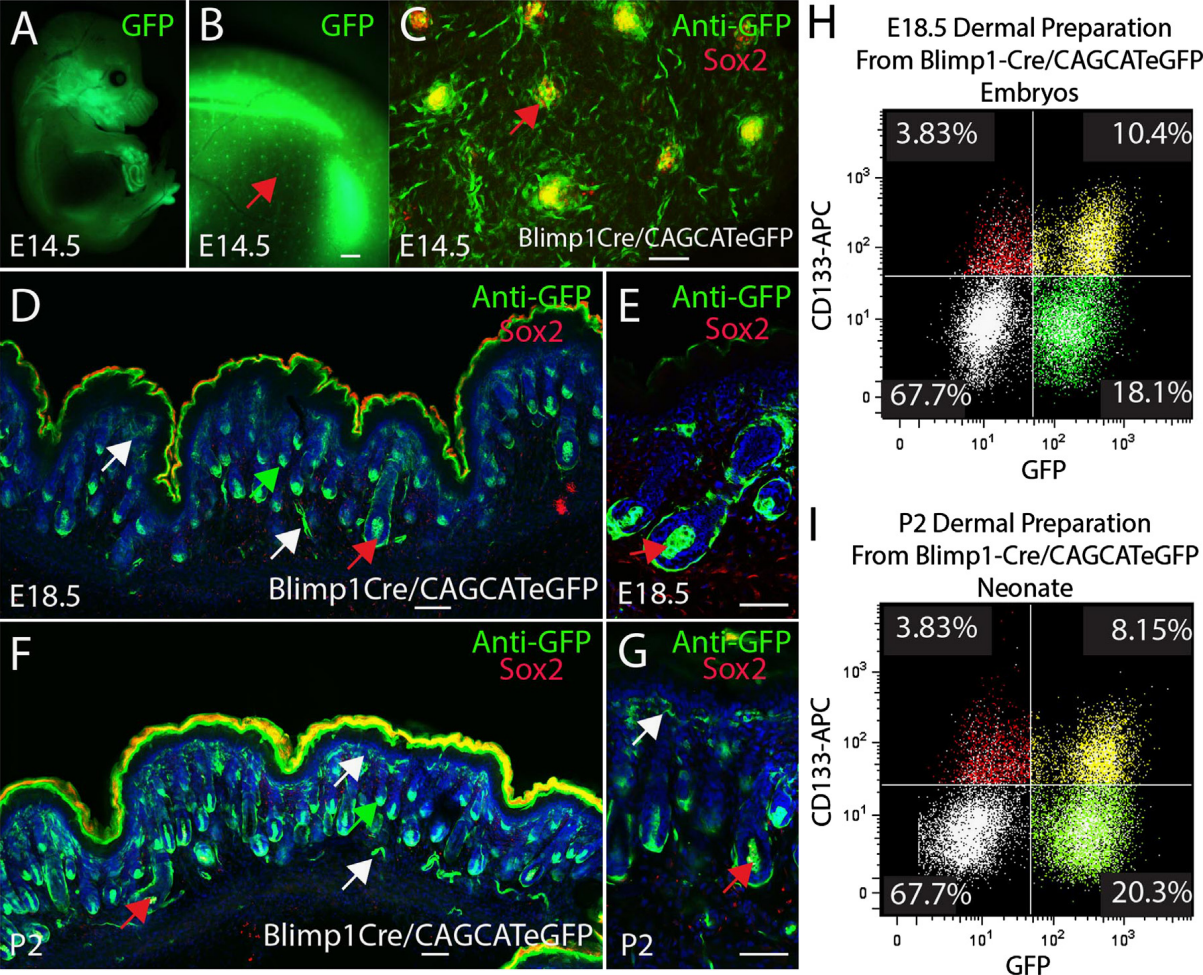
Blimp1 expression defines the dermal papillae lineages (A–G). Cryosections of E14.5-P2 skin from Blimp1Cre/CAGCATeGFP double transgenic mice immunostained for GFP or Sox2. Guard hairs were GFP positive in whole mount embryos (A–B) and co-expressed Sox2 in newly forming dermal papillae, as visualised in whole mount skin preparations (red arrows) (C). The lineages derived from Blimp1 expressing cells were restricted to the dermal papilla (red and green arrows) and skin vasculature (white arrows) (D–G). (H, I) Flow cytometry of single cell suspension of E18.5 (H) and P2 (I) dermis showing co-expression of the dermal papilla marker CD133 and GFP in Blimp1Cre/CAGCATeGFP double transgenic mice. Cells in each quadrant are labelled with a different colour for ease of visualisation. Data are representative of at least *N*=3 biological replicates. Scale bars: (B) 1 mm (C–G) 100 μm.

Blimp1+ cells have previously been shown to give rise to mature DP, the dermal sheath, and the upper (papillary) dermis (Robertson et al., 2007). We therefore investigated whether Sox2+ and Sox2− DP share a common Blimp1+ precursor. We crossed Blimp1Cre mice with CAGCATeGFP mice and then examined the location of GFP-positive cells, as they are derived from cells expressing Blimp1Cre. GFP was expressed in cells of the dermal papilla of guard hair follicles at E16.5 (Fig. 2A–C, red arrows) and in awl/auchene (red arrows) and zigzag (green arrows) hair follicles at E18.5 and P2 (Fig. 2D–G). In addition, Blimp1Cre cells gave rise to cells in the papillary region of the dermis as well as to the vasculature in the hypodermis (Fig. 2D, F, G, white arrows).

Flow cytometry showed that 67–75% of CD133+ cells in E18.5 and P2 dermis were also GFP+ (Fig. 2H and I), indicating that Blimp1+ cells give rise to the majority of DP cells. The proportion of GFP-positive cells was higher than in the sorts of Blimp1GFP skin (Fig. 1K and L). This is as expected, given that in the Blimp1Cre crosses GFP was expressed both by cells that express endogenous Blimp1 and by the progeny of Blimp1-positive cells.

The lineage relationship between dermal papilla, dermal sheath, and cells in the papillary region has not been clearly defined. Our results suggest that these cell types share a common embryonic origin and may be distinct from other fibroblast populations in the dermis.

### Sox2 is dispensable for dermal papilla formation and hair follicle morphogenesis

**Fig. 3:**
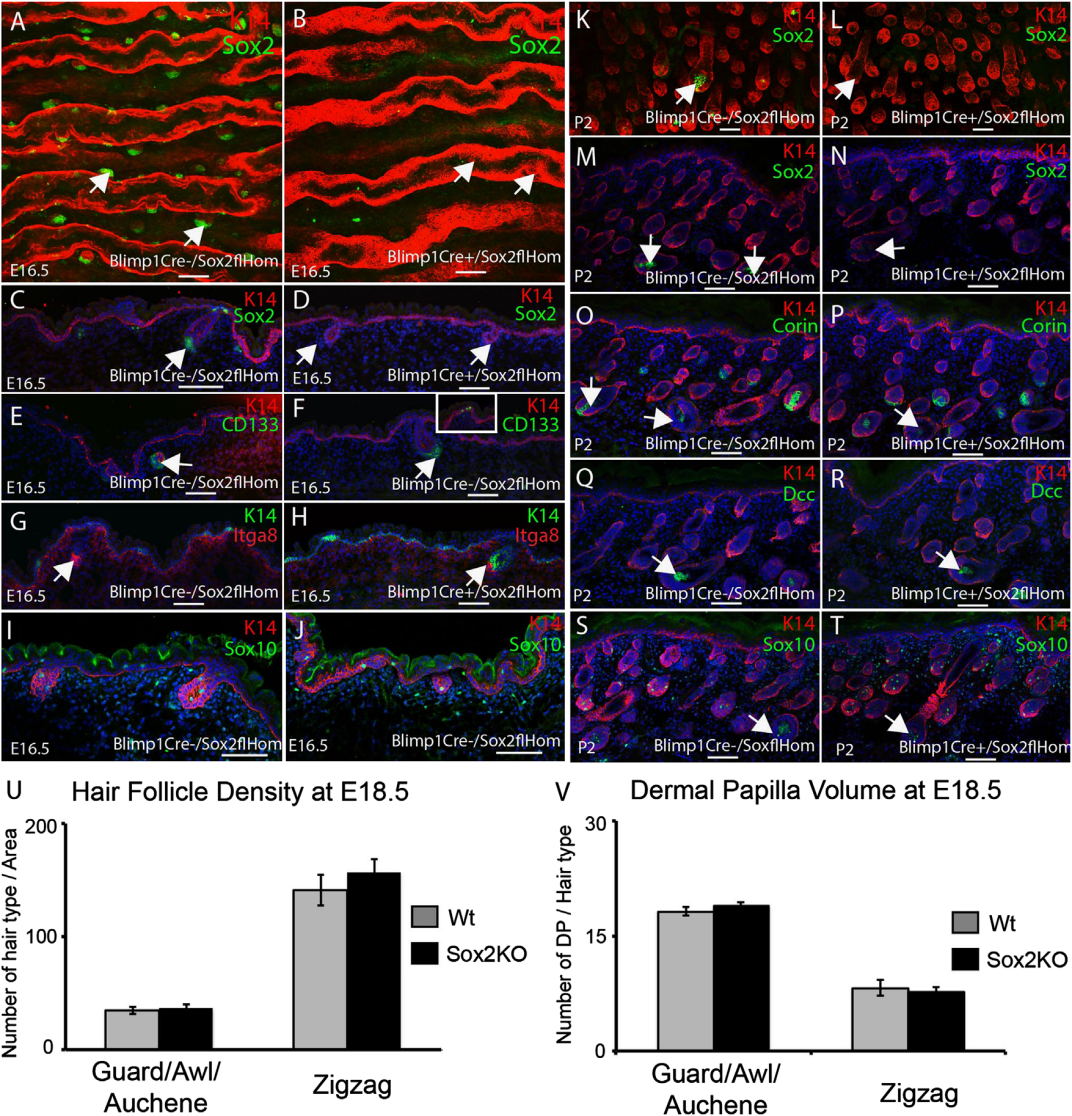
Sox2 is dispensable for dermal papilla formation and hair follicle morphogenesis. (A–B, K, L) Wholemounts of E16.5 and P2 skin from Blimp1Cre−/Sox2flHom (A, K) and Blimp1Cre+/Sox2flHom (B, L) mice labelled with the antibodies indicated. Guard/awl/auchene hair follicles are marked with arrows. (C–J, M–T) Cryosections of E16.5 and P2 skin from Blimp1Cre−/Sox2flHom (C, E, G, I, M, O, S) and Blimp1Cre+/Sox2flHom (D, F, H, J, N, P, R, T) mice immunostained with the antibodies indicated. Arrows mark guard/awl/auchene hair follicles. Scale bars: 100 μm. (U, V) Quantitation of hair follicle density (U) and number of cells per DP (V) in Blimp1Cre-/Sox2flHom (Wt) and Blimp1Cre+/Sox2flHom (Sox2KO) E18.5 embryonic skin. *N*=3 embryos per genotype. The number of cells per DP of 11 hair follicles of each type was counted per embryo.

Our data on Blimp1 expression indicated that expression of Cre under the control of Blimp1 transcriptional regulatory elements could be used to ablate gene expression in all DP at the earliest stages of dermal papilla morphogenesis. Blimp1Cre mice were therefore crossed with Sox2fl/fl mice. Blimp1Cre+/Sox2fl/fl mice were born at the expected Mendelian ratio but died shortly after birth, probably because of Sox2 deletion in the brain. Analysis of Sox2 expression in Blimp1Cre/Sox2fl/fl wholemounts and thick cryosections of skin confirmed efficient ablation of Sox2 in the DP of E16.5 follicles, in contrast to control littermates (Fig. 3A–D).

Expression of the DP markers CD133, Itga8, Corin and two specific markers of guard/awl/auchene DP, Dcc and Sox10 (Enshell-Seijffers et al., 2008; Driskell et al., 2009), was unaffected by Sox2 deletion (Fig. 3E–J, O–T and data not shown). At P2 dermal papilla and hair follicle development was unaffected by the absence of Sox2 (Fig. 3K–N). Deletion of Sox2 did not affect the number of guard/awl/auchene hair follicles (Fig. 3U) or the number of cells per DP (Fig. 3V) at E18.5.

### Sox2 is required for maintenance of postnatal awl/auchene hair follicles

**Fig. 4:**
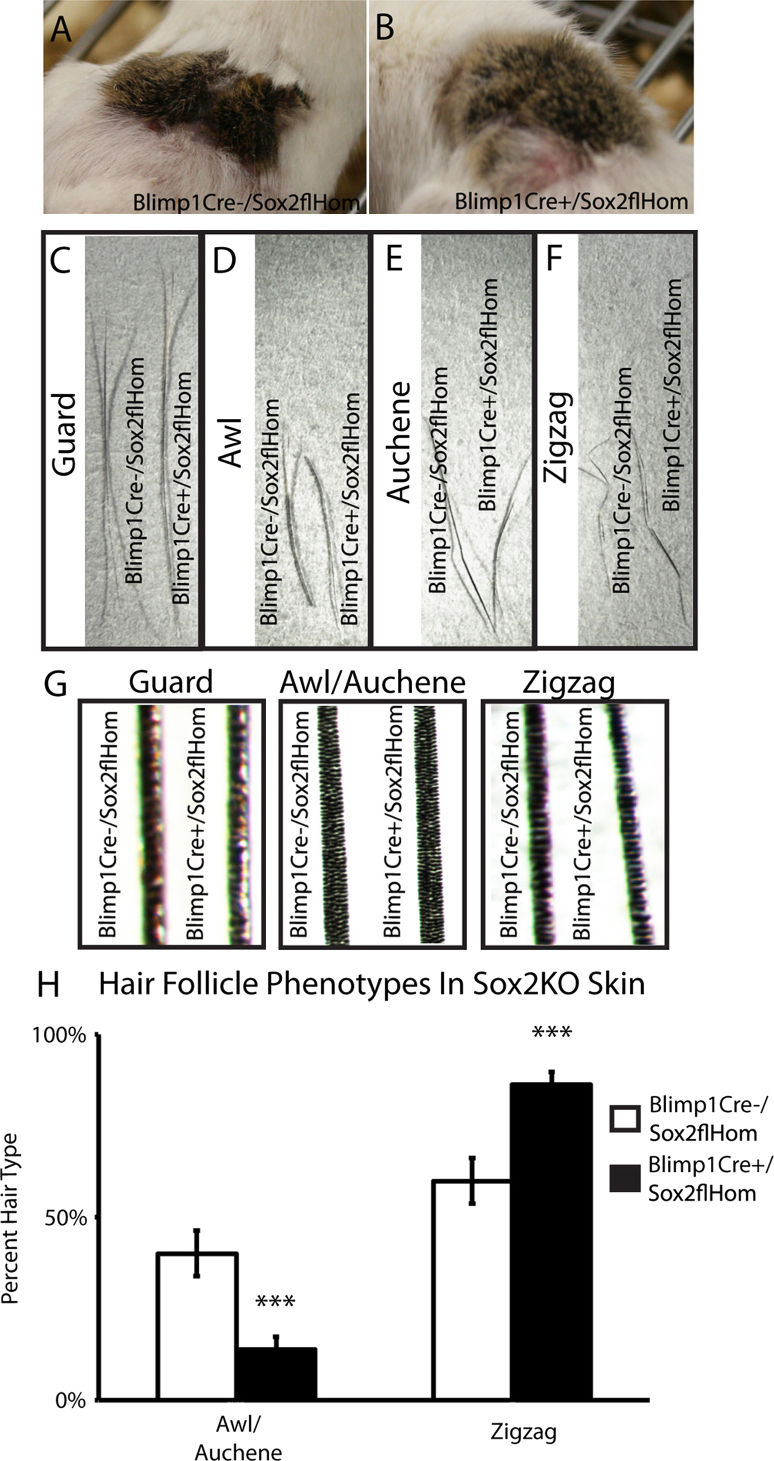
Sox2 is required for postnatal awl/auchene hair follicles. (A–B) E18.5 embryonic skin from Blimp1Cre-/Sox2flHom (A) and Blimp1Cre+/Sox2flHom (B) mice grafted on the back of NSG mice. (C–F) Grafts were harvested at 10 weeks. Guard (C), awl (D), auchene (E) and zigzag (F) hair follicle length was the same in Sox2+ and Sox2− grafts. (G) Medulla cell width was not affected by Sox2 deletion. (H) Quantitation of hair follicle types distinguished on the basis of medulla cell width and hair follicle length (biological replicates *n*=4). ***P<0.003.

To investigate whether Sox2 plays a role in the postnatal maintenance of hair follicles we grafted E18.5 Blimp1Cre/Sox2fl/fl and control littermate skin onto NSG immunodeficient mice. At the macroscopic level, 10 week old wild type and Sox2 null grafts were indistinguishable (Fig. 4A and B). The length and number of kinks in plucked hair follicles were not affected by the loss of Sox2, regardless of hair follicle type (Fig. 4C–F) (Driskell et al., 2009). There was also no difference in the number of medulla cells contained in the different hair follicle types (Fig. 4G). The total number of hairs and proportion of guard hairs were unaffected by lack of Sox2 (data not shown). In wild type skin approximately 30% of follicles are awl/auchene, while 67% are zigzag (Duverger and Morasso, 2009). There was a 3-fold decrease in the percentage of awl/auchene hair follicles when Sox2 expression was ablated and a corresponding increase in zigzag follicles (Fig. 4H). These results indicate that Sox2 expression influences the ratio of awl/auchene hair follicles in mice.

### Sox2 expression in Merkel cells

**Fig. S1:**
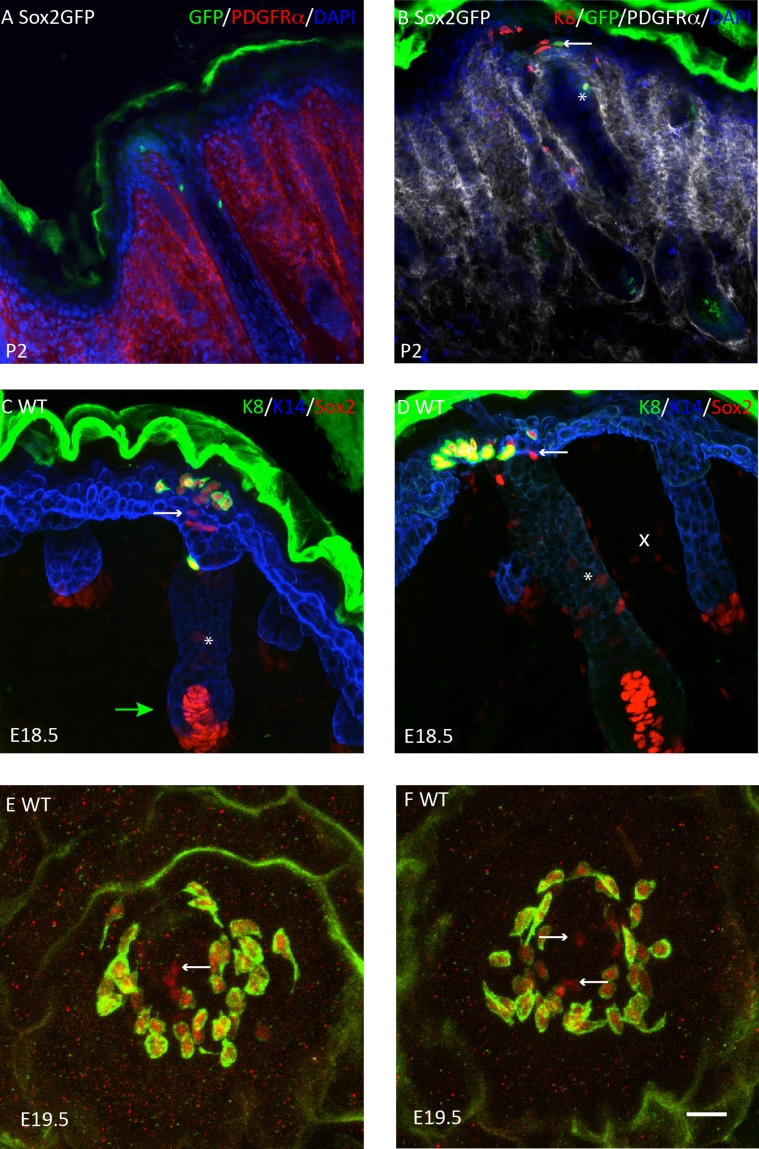
Characterisation of Sox2+ K8− Merkel cells. Horizontal wholemounts (A–D) showing (A, B) Sox2GFP expression (green) or (C, D) endogenous Sox2 (red) in skin sections labelled with antibodies to PDGFRa (red in A; white in B), K8 (green in C, D) or K14 (blue in C, D) and counterstained (A, B) with DAPI (blue). Sox2+K8+PDGFRa− cell is indicated by white arrow in B. Green arrow in C shows dermal sheath. White arrows in C, D show K8+K14+Sox2− cells. At E18.5 some weakly Sox2-positive cells are seen in the dermis (x) and around the hair follicles in the bulge area (*). These could potentially be nerve cells. (E, F) Wholemounts of E19.5 wild type touch domes immunolabelled for Sox2 (red) and K8 (green). Arrows show Sox2+K8− Merkel cells. Scale bar: 10 mm.

**Fig. 5:**
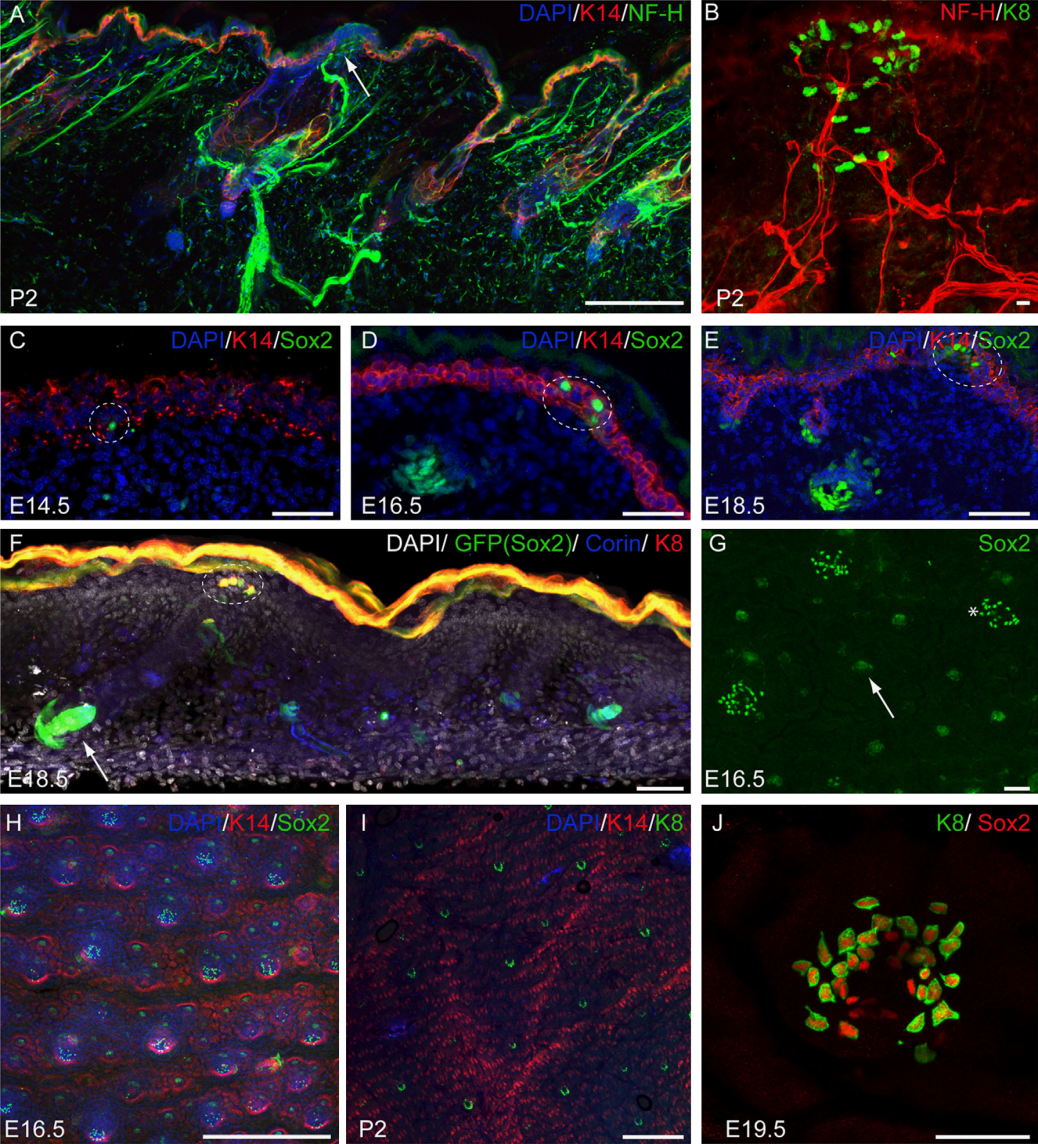
Sox2 is expressed by Merkel cells. (A) Visualisation of P2 back skin innervation in horizontal wholemount immunostained for NF-H (nerves), K14 (epidermal basal layer) and DAPI (nuclear counterstain). Arrow indicates site of nerve-Merkel cell-keratinocyte interaction. (B) Visualisation of single touch dome innervation in horizontal wholemount of P2 back skin immunostained for K8 (Merkel cells) and NF-H. (C–E) Back skin sections (E14.5–18.5) immunostained for Sox2, K14 and DAPI. (F) Horizontal wholemount of E18.5 back skin from Sox2GFP mouse immunostained for GFP, Corin, K8 and DAPI. (G) E16.5 back skin wholemount immunostained for Sox2. Sox2-positive cells within the epidermis are demarcated with dashed lines (C–F); arrows indicate Sox2-positive DP (F, G); asterisk indicates Merkel cell cluster (G). (H) E16.5 back skin wholemount immunostained for Sox2, K14 and DAPI showing distribution of Merkel cell clusters. (I) P2 back skin wholemount immunostained for K8, K14 and DAPI; note similar distribution of K8−positive cells to Sox2-positive cells (H). (J) E19.5 back skin wholemount showing a single touch dome immunostained for Sox2 and K8. Note K8+Sox2+ and K8−Sox2+ cells. Scale bars: 500 μm (H–I); 100 μm (A, B); 50 μm (C–G, J).

Merkel cells reside in the epidermal basal layer, directly linked to nerve cells containing NF-H positive neurofilaments (Fig. 5A and B). A few Sox2-positive Merkel cells were identified as early as E14.5 of embryonic skin development, with the majority appearing at E15.5 (Fig. 5C–E). At E18.5 the touch domes were fully developed, with Merkel cells being located in the epidermal basal layer around the guard hairs (Fig. 5F and G). Merkel cells were not found in association with awl/auchene or zigzag hairs (Fig. 5F and G). At E18.5 some weakly Sox2-positive cells were seen in the dermis and around the hair follicles in the bulge area, which could potentially be nerve cells (Fig. S1).

**Fig. S2:**
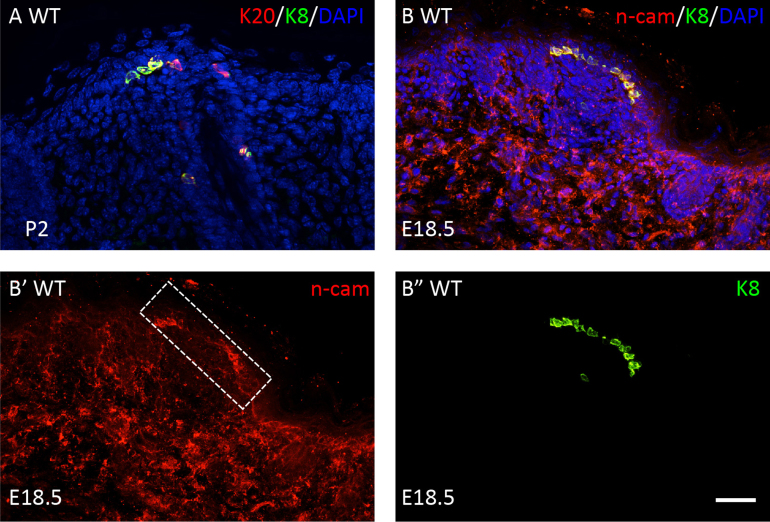
Co-expression of Merkel cell markers. E18.5 wild type skin was immunolabelled for (A) K20 (red) and K8 (green) with DAPI counterstain (blue) or (B–B″) for n-cam (red), K8 (green) and DAPI (blue). (B–B″) show the same field. Scale bar: 100 mm.

Immunostaining for Sox2 and the Merkel cell marker Keratin 8 (K8) (Haeberle et al., 2004) revealed that whereas all K8-positive cells expressed Sox2, a small population of Sox2-positive cells were K8-negative at both E16.5 and E19.5 (Fig. 5H–J; Fig. S1E and F). All K8+ cells expressed K20 (Eispert et al., 2009), whereas heterogeneity in N-cam expression was observed (Fig. S2). Sox2+K8− cells expressed K14 and were negative for the fibroblast marker PDGFRα, establishing that they were epidermal and not dermal sheath cells (Fig. S1A–D). Heterogeneity of Merkel cells has been observed previously (Eispert et al., 2009; Tachibana et al., 1997) and we speculate that Sox2-positive, K14-positive, K8-negative cells resident in the epidermis at E16.5 and E19.5 are Merkel cells in the initial phase of differentiation (Van Keymeulen et al., 2009).

### Sox2 ablation results in a decrease in Merkel cell number without affecting epidermal homoeostasis or guard hair patterning

**Fig. 6:**
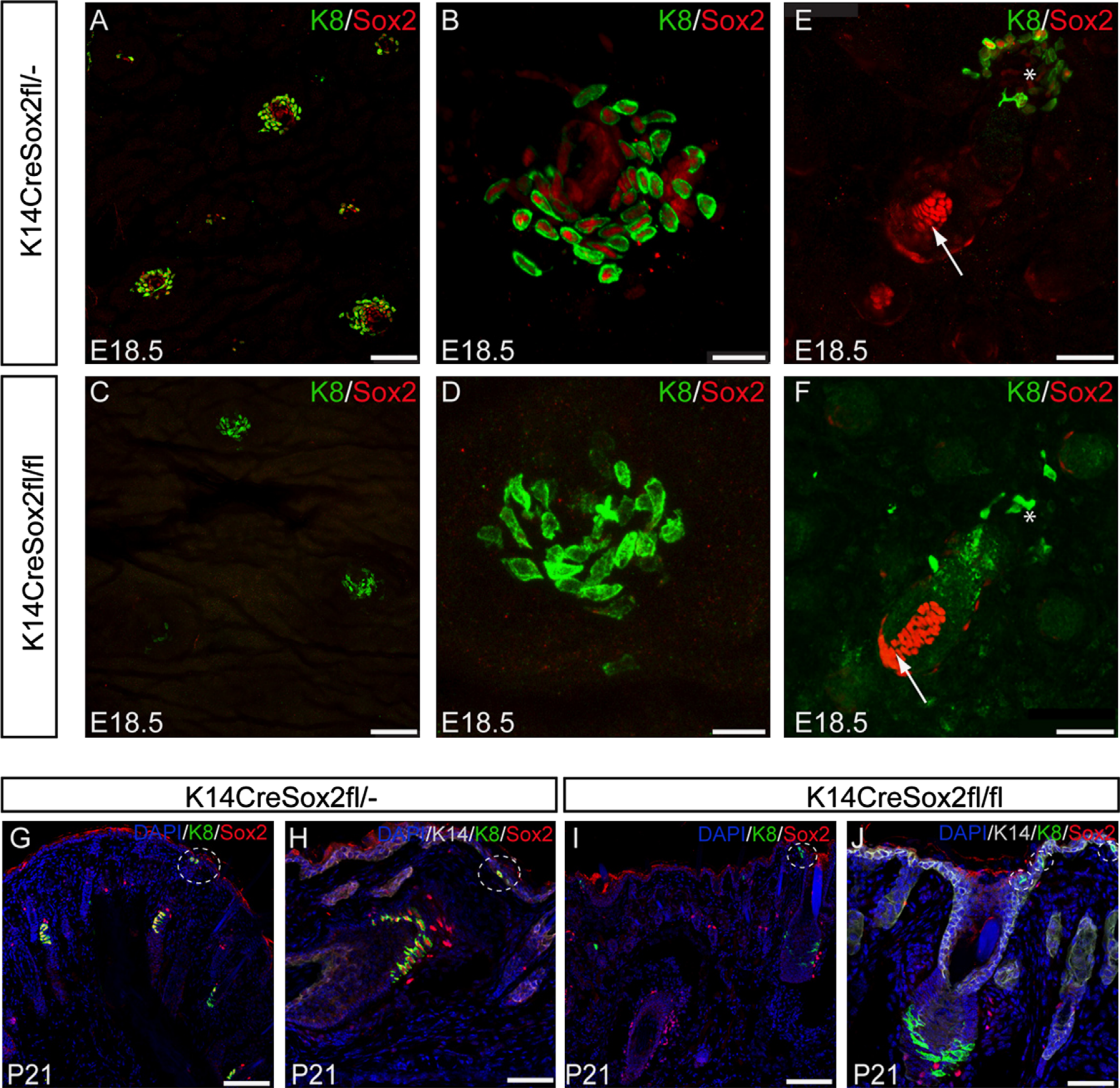
Sox2 ablation in Merkel cells via K14Cre. (A–D) E18.5 K14CreSox2fl/− (A, B) and E18.5 K14CreSox2fl/fl (C, D) back skin wholemounts immunostained for K8 and Sox2 and visualised from the epidermal side. (E–F) E18.5 K14CreSox2fl/− and K14CreSox2fl/fl back skin wholemounts immunostained for K8 and Sox2 and visualised from the dermal side. (G–J) Back skin sections from P21 K14CreSox2fl/− and K14CreSox2fl/fl mice immunolabelled with the antibodies shown. Asterisks and dashed circles demarcate Merkel cells; arrows mark dermal papillae. Scale bars: 100 μm (A, C); 50 μm (E–J); 25 μm (B, D).

In order to delete Sox2 we used K14Cre, which has previously been used to delete Atoh1/Math1 in Merkel cells (Van Keymeulen et al., 2009; Morrison et al., 2009). K14 is first expressed in the epidermal basal layer at E9.5 (Byrne et al., 1994; Zhou et al., 1995), prior to the onset of Sox2 expression (Fig. 5C–E). K14CreSox2fl/fl mice did not display any gross abnormalities and were born in the expected Mendelian ratios. Immunostaining of back skin wholemounts and whisker pad histological sections for Sox2 and K8 revealed that Sox2 was successfully deleted, with only a small number of Sox2-positive cells detectable at E16.5 (data not shown). In spite of Sox2 deletion, some Merkel cells still differentiated (Fig. 6A–D and G–J). As expected, the dermal papillae, which lack K14, still expressed Sox2 (Fig. 6E and F) and Sox2 deletion in Merkel cells did not affect the ratio of the different hair follicle types. Conversely, Sox2 deletion via Blimp1Cre did not result in loss of Sox2 from Merkel cells (Fig. 3F). Skin of control, K14CreSox2fl/^−^, littermates had Sox2+ Merkel cells (Fig. 6A, B, G, H).

**Fig. S3:**
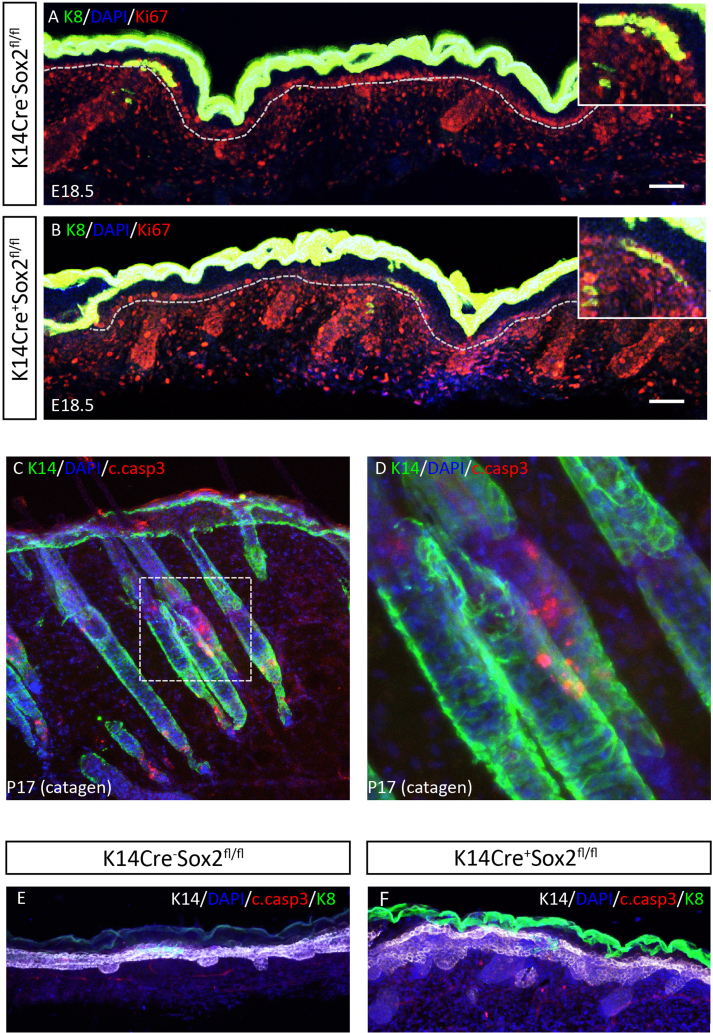
Proliferation and apoptosis on epidermal deletion of Sox2. (A, B) Back skin sections immunolabelled for Ki67 (red) and K8 (green) with DAPI nuclear counterstain (blue). Dashed lines marks boundary between interfollicular epidermis and dermis. Inserts show higher magnification views of selected areas. (C, D) Detection of cleaved Caspase 3 (c.casp3; red) in wild type catagen hair follicles co-stained for K14 (green) with DAPI nuclear counterstain (blue). This provides a positive control for the antibody to cleaved Caspase 3. (D) shows higher magnification view of boxed area in (C). (E, F) Cleaved Caspase 3 (red) is not detected in Merkel cells. Sections were also immunolabelled for K14 (white) and K8 (green) with DAPI nuclear counterstain. Note that K8 staining of cornified layers is non-specific (A, B, F). Scale bars: 100 mm (A, B, E, F); 10 mm (C).

**Fig. 7:**
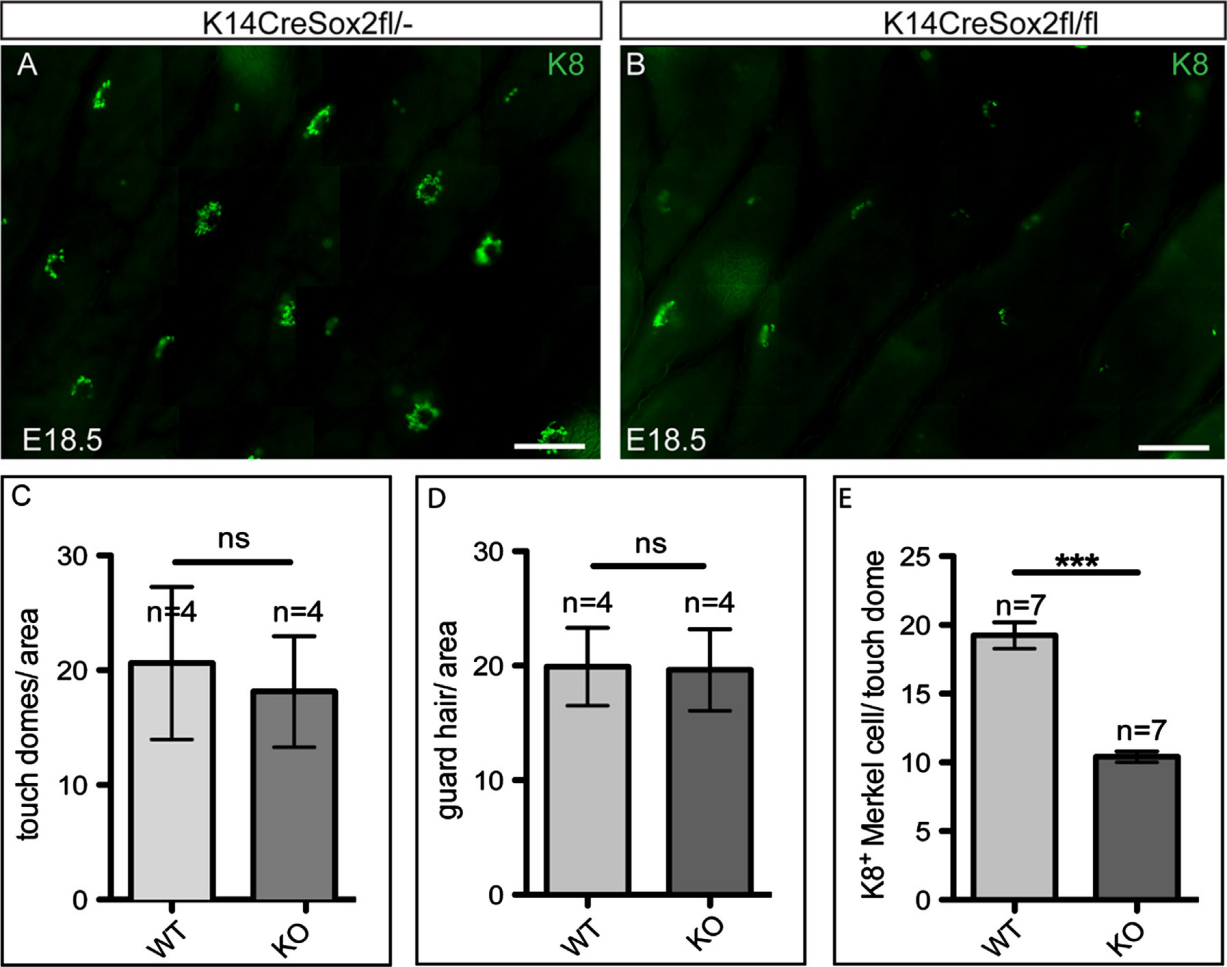
Deletion of Sox2 results in reduced number of Merkel cells. (A, B) Immunofluorescence labelling of Merkel cells with antibody to K8 in back skin wholemounts of K14CreSox2fl/− (A) and K14CreSox2fl/fl (B) mice, visualised from the epidermal side. (C) Quantification of number of touch domes per area (microscopic field) of back skin (*n*=4 biological replicates). (D) Quantification of guard hair number per area (microscopic field) of back skin (*n*=4 biological replicates). (E) Quantification of number of K8+ Merkel cells/per touch dome (*n*=7 biological replicates, 30 touch domes per replicate). Scale bars: 50 μm.

To investigate whether Sox2 deletion had any effect on the number of Merkel cells per touch dome, back skin wholemounts from K14CreSox2fl/^−^ and K14CreSox2fl/fl mice were immunostained for K8 (Fig. 7A and B). Deleting Sox2 did not alter the number of touch domes per unit area of skin (Fig. 7C), nor the number of guard hairs (Fig. 7D). However, there was a significant reduction in the number of K8-positive Merkel cells per touch dome (30 touch domes scored per skin sample; 7 biological replicates per genotype; *p*<0.0001) in K14CreSox2fl/fl skin (Fig. 7E). This did not correlate with any change in cell density within the touch domes (Fig. 6B and D). We could not detect any proliferative, Ki67-positive Merkel cells regardless of whether or not Sox2 was deleted (Fig. S3A and B). Staining for cleaved Caspase 3 established that the reduced number of Merkel cells on Sox2 deletion did not reflect increased apoptosis (Fig. S3C–F). We therefore conclude that Sox2 plays a role in regulating Merkel cell differentiation.

**Fig. 8:**
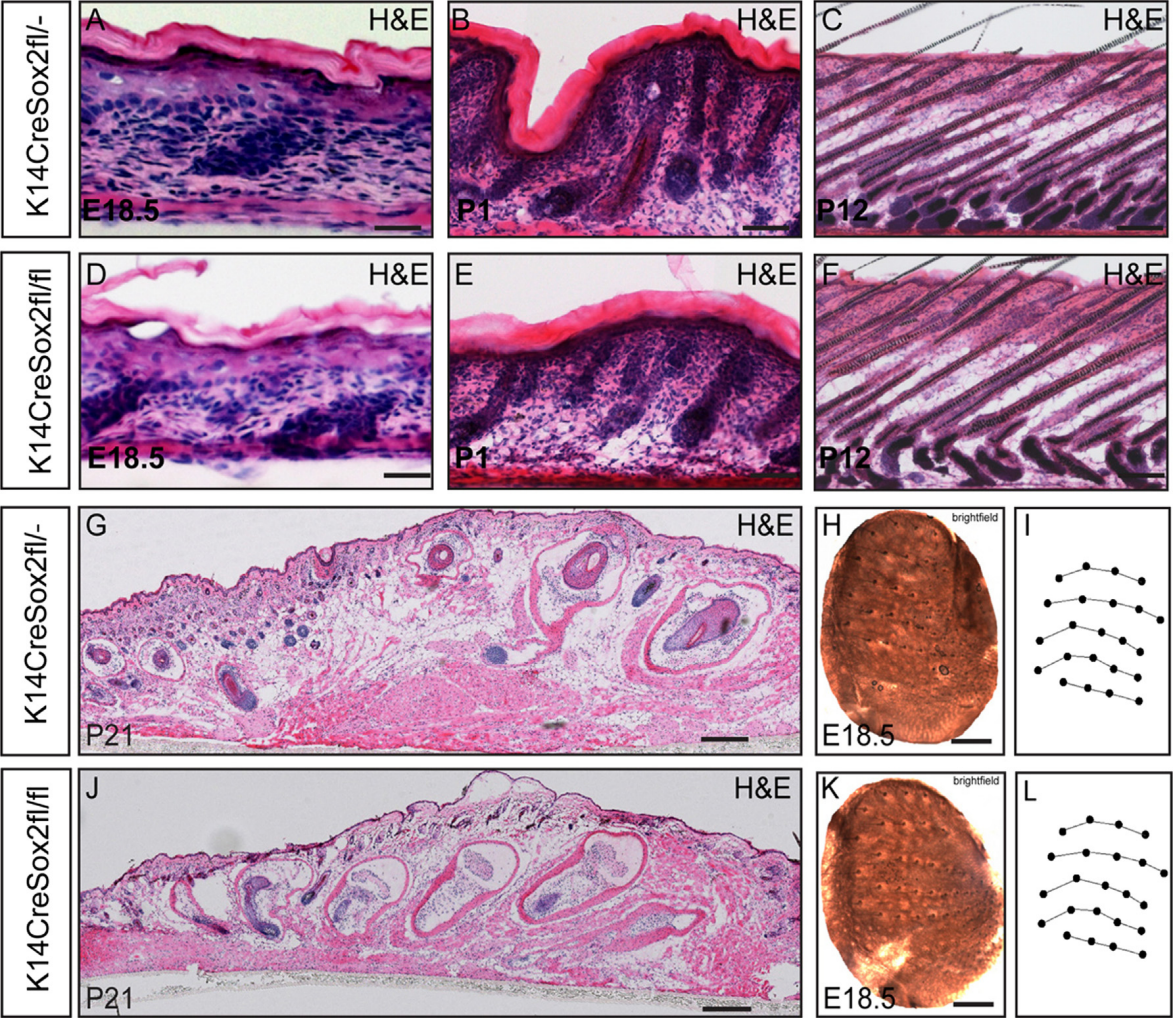
Epidermis and whisker pad morphology are unaltered on Sox2 deletion. (A–F) H&E stained sections of back skin from K14CreSox2fl/− (A–C) and K14CreSox2fl/fl (D–F) mice at the stages shown. (G, J) H&E stained sections of P21 whisker pad skin sections from K14CreSox2fl/− and K14CreSox2fl/fl mice. (H–L) unstained wholemounts of whisker pads from K14CreSox2fl/− (H) and K14CreSox2fl/fl (K) mice. (I, L) Schematic representation of whisker patterns in (H, I). Scale bars: 1000 μm (H, K); 500 μm (G, J); 25 μm (A–E).

In spite of the reduction in Merkel cell number, epidermal homoeostasis was unaltered in K14CreSox2fl/fl mouse back skin during development and in adult life (Fig. 8A–F). Tissue homoeostasis in P21 K14CreSox2fl/fl whisker pads was also normal when compared with K14CreSox2fl/^−^ controls (Fig. 8G and J). When we compared patterning of guard hairs (vibrissae) in the whisker pads of E18.5 wild-type and K14CreSox2fl/fl mice, we found no effect of Sox2 deletion (Fig. 8H, I, K, L).

### Sox2 is not required for touch dome innervation

**Fig. 9:**
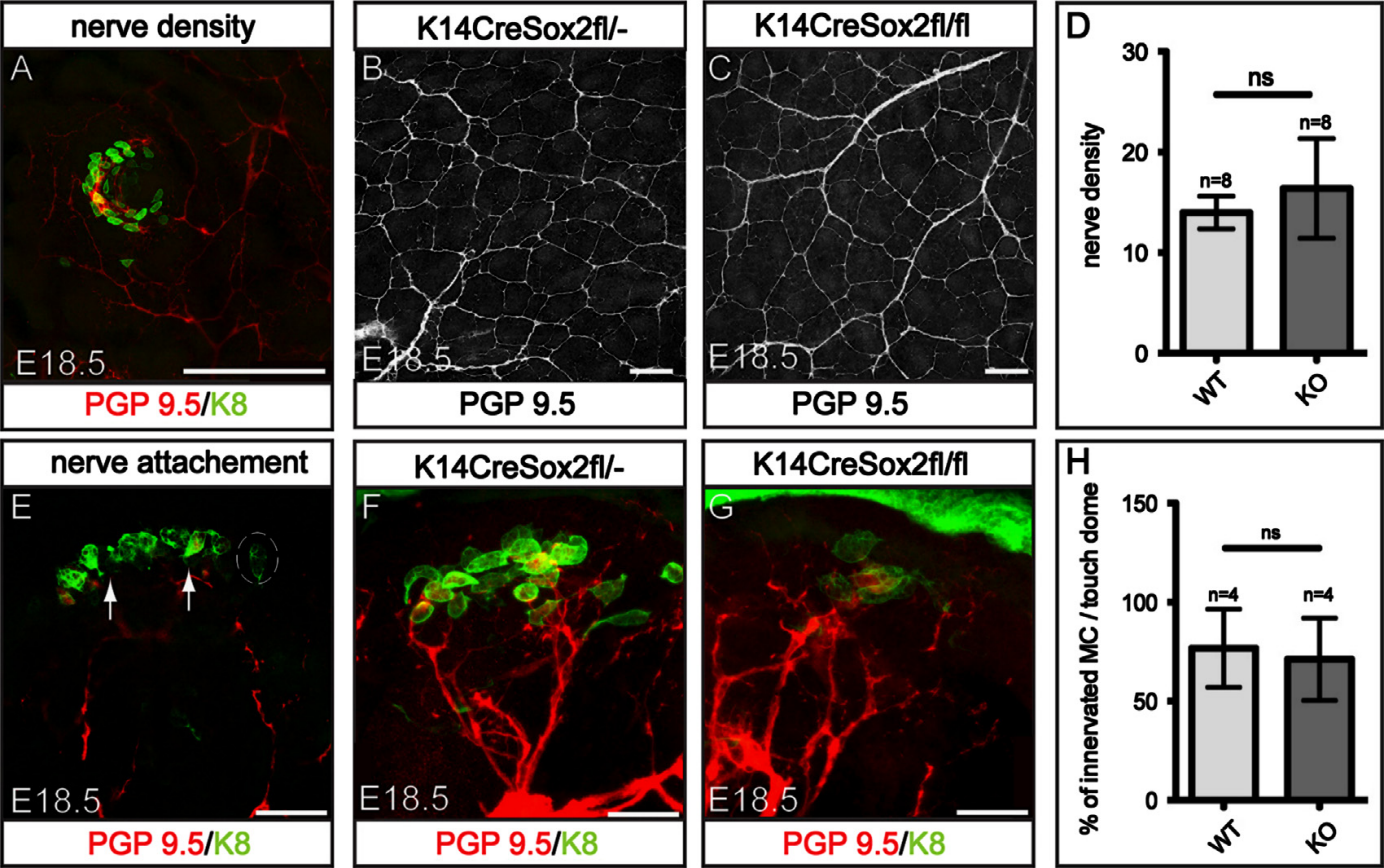
Touch dome innervation on Sox2 deletion. (A) E18.5 K14CreSox2fl/- back skin wholemount immunostained for K8 and PGP9.5 and visualised from the epidermal side, showing PGP9.5+ nerves interacting with touch domes. (B, C) E18.5 back skin wholemounts from K14CreSox2fl/− and K14CreSox2fl/fl mice immunostained with PGP9.5 to illustrate nerve density. (D) Quantification of nerve density in K14CreSox2fl/− and K14CreSox2fl/fl mice (*n*=8 biological replicates). (E) Single plane confocal image of horizontal wholemount from K14CreSox2fl/− E18.5 back skin immunostained with PGP9.5 and K8. Arrows indicate nerve–Merkel cell interaction; encircled Merkel cells are not associated with nerves. (F, G) Confocal z-stacks showing touch dome innervation in K14CreSox2fl/− and K14CreSox2fl/fl mouse back skin. Horizontal wholemounts were immunostained for PGP9.5 and K8. (H) Quantification of % of innervated Merkel cells per touch dome in K14CreSox2fl/− and K14CreSox2fl/fl mice (*n*=4 biological replicates). Scale bars: 100 μm (A–C); 25 μm (E–G).

In order to investigate the effect of the decreased number of Merkel cells on skin innervation, we stained back skin whole mounts of E18.5 K14CreSox2fl/fl and wild-type littermate mice with an antibody to the neuronal marker PGP9.5 (Fig. 9A). Quantification of nerve density showed that there was no significant difference in nerve density between K14CreSox2fl/fl and K14CreSox2fl/^−^ skin (Fig. 9B–D).

We next prepared horizontal whole mounts (Driskell et al., 2012b) of E18.5 embryonic skin stained with PGP9.5 and K8 to visualise nerve–Merkel cell interactions. In both wild type and K14CreSox2fl/fl skin some Merkel cells within each touch dome did not interact directly with nerves (Fig. 9E). We quantitated the percentage of innervated Merkel cells per touch dome by acquiring z-stack images and analysing each plane. Analysis of 40 touch domes per mouse from E18.5 K14CreSox2fl/fl and wild type littermate skin (*n*=4 mice per genotype) did not reveal a significant difference (Fig. 9F–H). Therefore deleting Sox2 and thereby reducing Merkel cell number does not affect nerve density and touch dome innervation.

### Sox2-negative Merkel cells are capable of synaptic processes

**Fig. 10:**
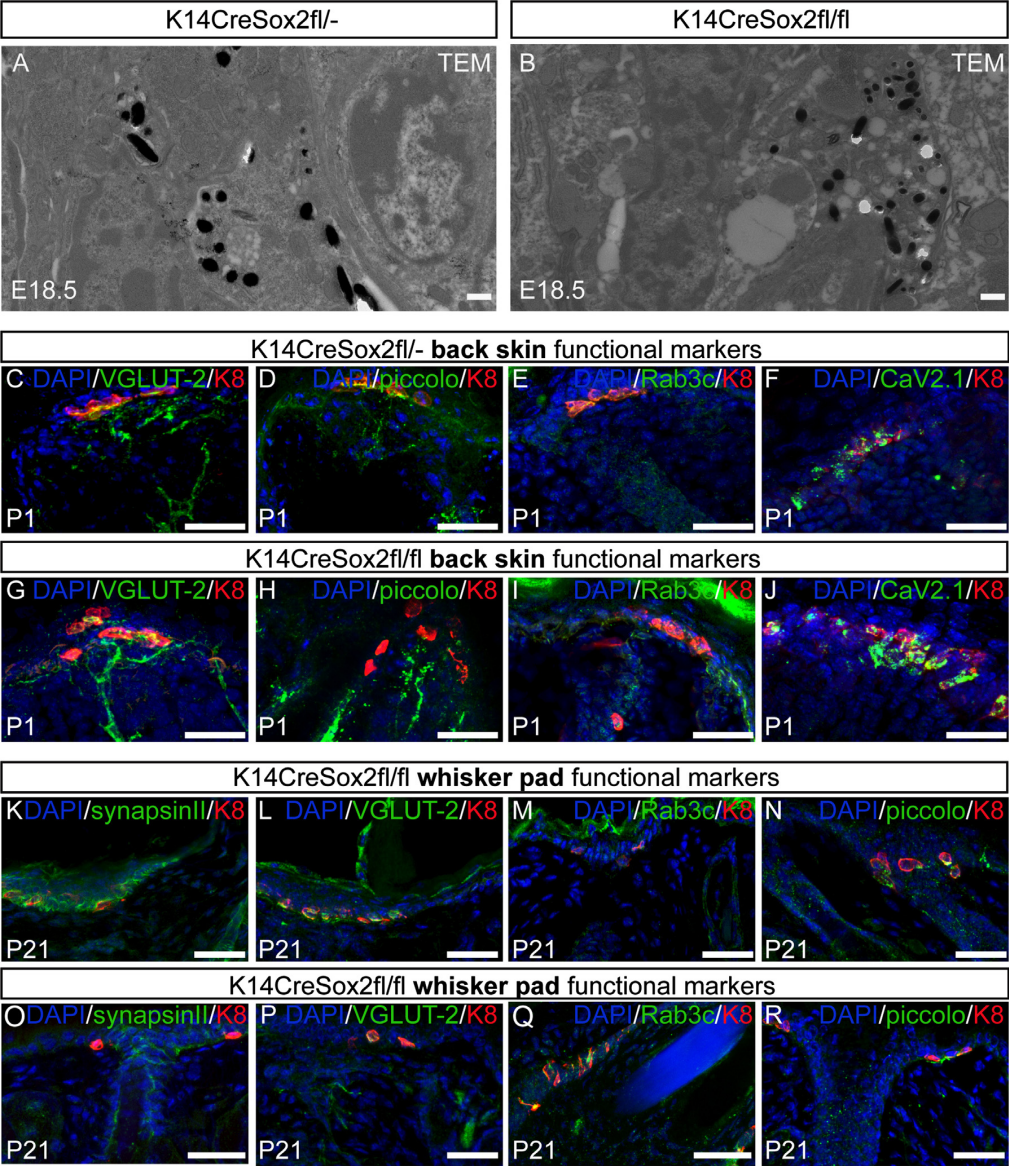
Sox2 −/− Merkel cells are capable of synaptic processes. (A–B) Transmission electron micrographs of K14CreSox2fl/− and K14CreSox2fl/fl back skin sections, showing synaptic vesicles. (C–J) Confocal images of back skin sections from K14CreSox2fl/− and K14CreSox2fl/fl mice immunostained with antibodies to VGLUT-2, piccolo, Rab3c or Cav2.1 and K8, counterstained with DAPI. (K–R) Confocal images of whisker pad sections from K14CreSox2fl/− and K14CreSox2fl/fl mice immunostained with antibodies to VGLUT-2, piccolo, Rab3c or synapsin II and K8, counterstained with DAPI. Scale bars: 50 μm (K–R); 25 μm (C–J); 500 nm (A, B).

Merkel cells are excitatory cells that carry out synaptic processes (Haeberle et al., 2004; Piskorowski et al., 2008). Transmission electron microscopy of E18.5 back skin revealed that in both K14CreSox2fl/fl and wild-type littermate mice small, electron dense synaptic vesicles were present in Merkel cells (Fig. 10A and B). Furthermore, expression of markers of synapse function, VGLUT-2, Rab3c, piccolo and Cav2.1, was unaffected by deletion of Sox2 in back skin (Fig. 10C–J) and in the whisker pads (Fig. 10K–R). We conclude that although Sox2 deletion leads to a decrease in the number of Merkel cells those cells that do differentiate show normal expression of a range of functional markers.

## Discussion

Sox2 has long been recognised as a marker of embryonic pluripotency (Takahashi and Yamanaka, 2006), and recent data show that Sox2 expressing cells are also important for maintenance of adult tissues. Arnold et al. (2011) found that ablating Sox2-positive cells in many epithelia, including tongue and stomach, led to disruption of tissue homoeostasis with consequent lethality. Nevertheless, they did not observe a phenotype in the epidermis. This is perhaps unsurprising, since only a tiny minority of epidermal cells, the Merkel cells, express Sox2. We have now investigated the consequences of deleting Sox2 in Merkel cells and DP cells.

Current approaches to modulating gene expression in the dermal papilla include driving Cre expression via dermal papilla specific or pan-fibroblast promoters (Enshell-Seijffers et al., 2008; Chen et al., 2012; Hamburg and Atit, 2012; Woo et al., 2012; Grisanti et al., 2013). We found that during hair follicle morphogenesis all follicle types express Blimp1 in the dermal condensate at E14.5, when the first hair follicles start to form. Blimp1Cre could therefore be used to ablate Sox2 expression in the dermal papilla. Although almost all DP cells expressed Blimp1 a few were negative. Further experiments are required to determine whether this reflects an underlying cellular heterogeneity within individual DP.

Ablation of Sox2 in the DP did not affect hair follicle morphogenesis but did modulate hair follicle type. Since the decrease in awl/auchene follicles was correlated with an increase in zigzag hairs, rather than a decrease in overall follicle number, it is likely that Sox2 specifies follicle subtype, rather than being required for maintenance of awl/auchene follicles. We have previously shown that Sox2-expressing cells are required for formation of awl/auchene follicles in skin reconstitution assays (Driskell et al., 2009) and our new data point to a key role for Sox2 in that function. Nevertheless, Sox2-positive cells do not inhibit zigzag hair formation and can contribute to both awl/auchene and zigzag DP (Driskell et al., 2012b). Sox2 expression is maintained in cultured DP from awl/auchene follicles, but is not induced in other DP cells, suggesting that Sox2-positive DP cells may represent a distinct cell lineage (Driskell et al., 2012b). When DP cells are cultured as spheres and then placed in skin reconstitution assays hair follicle type does not correlate with DP size as Sox2 positive cells form smaller spheres than Sox2-negative cells (Driskell et al., 2012b). However, during mouse skin development the number of DP cells per follicle does correlate with the size and shape of the hair follicles that develop (Chi et al., 2013).

Our results highlight a distinction between those genes that are required for core DP functions and those that specify the identity of the dermal papillae of different types of follicle (Rendl et al., 2005; Driskell et al., 2009). Examples of the first category of gene include beta-catenin, Noggin and Smo, since ablation either prevents dermal papilla formation or causes the DP to dissociate before hair follicles can develop fully (Woo et al., 2012). In the latter category of genes are Sox2, which specifies awl/auchene follicles (Fig. 4), and Sox18, which specifies zigzag follicles (James et al., 2003). It has recently been reported that Sox2 also controls hair length (Clavel et al., 2012), as does beta-catenin (Enshell-Seijffers et al., 2010). We did not observe the same effect, but this could be due to the differences in the assay conditions or timing of Sox2 ablation. It will be interesting to examine the extent to which the different gene networks that regulate DP function interact.

When we ablated Sox2 in the epidermis via K14Cre, the number of Merkel cells per touch dome was reduced, confirming the recent studies of Bardot et al. (2013). Since Sox2 ablation had no effect on Merkel cell proliferation or apoptosis the reason for the reduction in cell number is most likely that fewer cells underwent differentiation. Ablation of Sox2 in Merkel cells had no discernible effect on hair follicle type and Sox2 deletion in the DP did not affect the number of Merkel cells, leading us to conclude that there are two functionally independent Sox2 expressing lineages in the skin. Given the lack of any obvious phenotypes out with the Merkel cells and DP it seems unlikely that Sox2 is required for the multi-lineage differentiation potential of SKPs, at least in vivo (Biernaskie et al., 2009).

It has been suggested previously that Merkel cells play a role in attracting nerve growth into the skin during embryonic development (Narisawa and Hashimoto, 1991; Scott et al., 1981, Lucarz and Brand, 2007). In support of this, deleting Atoh1 from Hoxb1+ cells (in the dermis and epidermis of body skin) results in exuberant branching of touch dome afferents (Maricich et al., 2009). However, in spite of the decrease in Merkel cell number on deletion of Sox2 there was no effect on overall skin nerve density, and sufficient Merkel cells remained to allow normal touch dome innervation. Our results are in agreement with the observation that an increase in the number of Merkel cells resulting from deletion of Ezh1 and Ezh2 does not alter skin innervation (Bardot et al., 2013). Furthermore, Doucet et al. (2013) have shown that it is the touch dome keratinocytes rather than differentiated Merkel cells that are required for innervation.

Sox2 was not required for expression of synaptic and voltage-gated signalling markers or formation of dense core synaptic vesicles in Merkel cells. Given that Sox2 is a well-established neural transcription factor, we speculate that other neural transcription factors are capable of compensating for the loss of Sox2 and maintaining expression of sufficient levels of synaptic proteins for synaptic vesicles to form. Indeed Sox2, which is repressed by the Polycomb complex (Bardot et al., 2013), acts as a positive regulator of the key transcription factor Atoh1 (Maricich et al., 2009).

In conclusion, we have shown that Sox2 plays a role in Merkel cell differentiation and in specifying dermal papilla subtypes. Sox2 thus plays a role in lineage commitment of both epithelial and mesenchymal cells within the skin.

## Footnotes

Appendix A Supplementary data associated with this article can be found in the online version at doi:10.1016/j.ydbio.2013.08.004.
